# Update: ACIP Recommendations for the Use of Quadrivalent Live Attenuated Influenza Vaccine (LAIV4) — United States, 2018–19 Influenza Season

**DOI:** 10.15585/mmwr.mm6722a5

**Published:** 2018-06-08

**Authors:** Lisa A. Grohskopf, Leslie Z. Sokolow, Alicia M. Fry, Emmanuel B. Walter, Daniel B. Jernigan

**Affiliations:** ^1^Influenza Division, National Center for Immunization and Respiratory Diseases, CDC; ^2^Battelle Memorial Institute, Atlanta, Georgia; ^3^Duke University School of Medicine, Durham, North Carolina.

Intranasally administered live attenuated influenza vaccine (LAIV) was initially licensed in the United States in 2003 as a trivalent formulation (LAIV3) (FluMist, MedImmune, LLC). Quadrivalent live attenuated influenza vaccine (LAIV4) (FluMist Quadrivalent, MedImmune) has been licensed in the United States since 2012 and was first available during the 2013–14 influenza season, replacing LAIV3. During the 2016–17 and 2017–18 influenza seasons, the Advisory Committee on Immunization Practices (ACIP) recommended that LAIV4 not be used because of concerns about low effectiveness against influenza A(H1N1)pdm09-like viruses circulating in the United States during the 2013–14 and 2015–16 seasons (*1*,*2*). On February 21, 2018, ACIP recommended that LAIV4 be an option for influenza vaccination of persons for whom it is appropriate for the 2018–19 season (*3*). This document provides an overview of the information discussed in the decision-making process leading to this recommendation. A description of methodology and data reviewed will be included in the background materials that will supplement the 2018–19 ACIP Influenza Recommendations, which will replace the 2017–18 ACIP influenza statement (*2*), and which will also contain guidance for the use of LAIV4.

Before the 2009 influenza A(H1N1) pandemic, three randomized trials noted superior relative efficacy of LAIV3 compared with trivalent inactivated influenza vaccine (IIV3) among children (*4*–*6*). However, LAIV4 demonstrated no statistically significant effectiveness against influenza A(H1N1)pdm09-like viruses among children aged 2 through 17 years in U.S. studies conducted during the 2013–14 and 2015–16 seasons (*7*–*12*), during which these viruses predominated. This lack of effectiveness was postulated as attributable to decreased replicative fitness of the influenza A(H1N1)pdm09-like viruses included in LAIV4 during those seasons (A/California/7/2009 for 2013–14 and A/Bolivia/559/2013 for 2015–16) (*13*). Investigations into the potential cause of this reduced effectiveness against influenza A(H1N1)pdm09 revealed that these LAIV viruses exhibited reduced replication in human nasal epithelial cells, compared with prepandemic influenza A(H1N1) LAIV viruses. For the 2017–18 season, a new influenza A(H1N1)pdm09-like virus (A/Slovenia/2903/2015) was included in LAIV4, replacing A/Bolivia/559/2013. However, LAIV4 was not recommended for use in the United States during 2017–18, and no U.S. effectiveness estimates were available.

## Methods

Data from three sources were presented to ACIP for discussion. These included 1) an analysis of the effectiveness of LAIV4 and inactivated influenza vaccines for the 2013–14 through 2015–16 seasons among children aged 2 through 17 years, using pooled data from five U.S. observational studies (*3*); 2) a systematic review of published literature regarding the effectiveness of LAIV3 and LAIV4 among children during the 2010–11 through 2016–17 seasons (*3*); and 3) a study conducted by the manufacturer that evaluated viral shedding and immunogenicity associated with LAIV4 containing the new influenza A(H1N1)pdm09-like virus (A/Slovenia/2903/2015) among U.S. children aged 24 months through <4 years (*14*).

## Summary of Data Reviewed

Review of LAIV effectiveness data for previous seasons in the United States confirms low to no significant effectiveness of LAIV against influenza A(H1N1)pdm09-like viruses. However, LAIV was generally effective against influenza B viruses and was of similar effectiveness to IIV against influenza A(H3N2) viruses. No effectiveness estimates were available for the current formulation of LAIV4 containing A/Slovenia/2903/2015 against influenza A(H1N1)pdm09-like viruses at the time of the review (*3*).

Data presented by the manufacturer indicated that the new LAIV4 influenza A(H1N1)pdm09-like virus, A/Slovenia/2903/2015, was shed by a higher proportion of children during days 4 through 7 following the first of 2 doses of vaccine. A/Slovenia/2903/2015 induced significantly higher antibody responses than its predecessor, A/Bolivia/559/2013. Seroconversion rates to A/Slovenia/2903/2015 were comparable to those obtained in response to prepandemic influenza A(H1N1) LAIV strains used during seasons in which the vaccine was observed to be effective against A(H1N1) influenza viruses (*14*).

The manufacturer also summarized information from previous presentations to ACIP concerning new candidate vaccine virus evaluation techniques that were employed in their investigation to identify the cause of low LAIV4 effectiveness, and how these techniques will be used going forward (*14*,*15*). Specifically, it was reported that two additional methods will be employed in the evaluation and selection of candidate vaccine viruses for inclusion in LAIV4, and these data will be shared each year with the Food and Drug Administration. Replicative fitness of candidate strains will be evaluated in human nasal epithelial cell culture. Previous methods using eggs and Madin-Darby canine kidney (MDCK) cell culture were found not to be predictive of replication of influenza A(H1N1)pdm09-like LAIV viruses in human cells. In addition, infectivity of vaccine viruses will be quantified using both 50% tissue culture infective dose (TCID_50_) and fluorescent focus assay (FFA), instead of FFA only. Whereas FFA measures expression of viral antigens on the cell surface and does not require multiple rounds of viral replication, TCID_50_ measures the spread of vaccine virus between cells through sustained replication cycles. Evaluation of influenza A(H1N1)pdm09-like viruses used in the 2013–14 (A/California/7/2009) and 2015–16 (A/Bolivia/559/2013) vaccines revealed that viral titers obtained via TCID_50_ were substantially lower than those obtained via FFA, indicating that these viruses were less able to sustain multiple rounds of replication. For A/Slovenia/2903/2015, the titers obtained via these two methods are similar and were comparable to those associated with prepandemic influenza A(H1N1) viruses with known efficacy (*15*).

## Discussion

Analyses of data from 2010–11 through 2016–17 indicate that LAIV was effective against influenza B viruses, and effectiveness against influenza A(H3N2) viruses was similar to that of inactivated influenza vaccines. During this period, LAIV was poorly effective among children aged 2 through 17 years against influenza A(H1N1)pdm09 viruses in the United States. Shedding and immunogenicity data provided by the manufacturer suggest that the new influenza A(H1N1)pdm09-like virus included in the current LAIV4, A/Slovenia/2903/2015, has improved replicative fitness over previous LAIV4 influenza A(H1N1)pdm09-like vaccine strains. However, no published effectiveness estimates for this formulation of the vaccine against influenza A(H1N1)pdm09 viruses were yet available because influenza A(H3N2) and influenza B viruses have predominated during the 2017–18 Northern Hemisphere season.

Effectiveness of influenza vaccines varies and is affected by many factors, including age and health status of the recipient, influenza type and subtype, prior influenza vaccination history, and degree of antigenic match between the vaccine and circulating viruses. It is possible that the vaccine effectiveness also differs among different individual vaccine products (for example, different IIVs); however, product-specific comparative effectiveness data are lacking for most vaccines. Although U.S. national influenza vaccination coverage among children did not decline substantially overall during the 2016–17 season (the first season in which it was recommended that LAIV not be used) (*3*), overall vaccination coverage remains suboptimal. Additional options for vaccination of children, including use of noninjectable vaccines such as LAIV4, might provide a means to improve coverage, particularly in school-based settings.

## Recommendation of the ACIP

For the 2018–19 U.S. influenza season, providers may choose to administer any licensed, age-appropriate influenza vaccine (IIV, recombinant influenza vaccine [RIV], or LAIV4). LAIV4 is an option for those for whom it is otherwise appropriate. No preference is expressed for any influenza vaccine product. ACIP will continue to review data concerning the effectiveness of LAIV4 as they become available. Providers should be aware that the effectiveness of the updated LAIV4 containing A/Slovenia/2903/2015 against currently circulating influenza A(H1N1)pdm09-like viruses is not yet known.
